# Racial and Ethnic Disparities in the Utilization and Outcomes of Catheter Ablation for Atrial Fibrillation and Flutter: a State-of-the-Art Review

**DOI:** 10.1007/s11886-026-02425-w

**Published:** 2026-10-06

**Authors:** Daniel Antwi-Amoabeng, T. David Gbadebo

**Affiliations:** 1 Division of Cardiovascular Disease, University of Alabama at Birmingham Heersink School of Medicine, Birmingham, AL 35294 USA; 2 Cardiovascular Care Center & Clinical Research Center, Conyers, GA 30012 USA

**Keywords:** Atrial fibrillation, Atrial flutter, Catheter ablation, Health equity, Healthcare disparities, Minoritized populations

## Abstract

**Purpose of Review:**

Catheter ablation (CA) has become a first-line option for rhythm control strategy for atrial fibrillation (AF) and atrial flutter (AFL). We synthesize recent evidence regarding racial and ethnic disparities in CA utilization in the United States and examine the structural mechanism driving them.

**Recent Findings:**

Black patients are 28 to 36% less likely to undergo CA for AF or AFL compared to White patients, with similarly reduced odds (22 – 35%) observed among Hispanic and Asian patients for AF. While subgroup analysis from clinical trials like CABANA demonstrate survival benefits of CA among minority cohorts, real-world data reveal that the very populations that stand to benefit the most are the least likely to receive CA. Public insurance models, upstream referral bottlenecks, geographic specialty deserts, and clinician-level implicit bias serve as primary structural drivers of these disparities.

**Summary:**

Racial and ethnic gaps persist in CA utilization and outcomes for both AF and AFL. AF/AFL prevalence is influenced by racial and ancestral biology while CA access disparity is driven by race as a social construct. These two phenomena require fundamentally different investigative frameworks and intervention targets.

## Introduction

Atrial fibrillation (AF) and atrial flutter (AFL) are the most prevalent sustained arrhythmias globally, affecting approximately 60 million individuals worldwide and presenting a significant healthcare burden with substantial morbidity, mortality, and economic costs [1]. Whereas AF/AFL burden is influenced by multiple cardiovascular risk factors and demographic variables, substantial racial and ethnic disparities exist in the diagnosis, management and clinical outcomes of these arrhythmias. AF has been reported at lower prevalence rates in Black populations despite Black patients bearing a higher burden of the traditional cardiovascular risk factors such as hypertension, heart failure, diabetes, and chronic kidney disease. This observation, termed the *AF paradox*, represents not only true epidemiologic differences, but also systemic management disparities and differential pathophysiological mechanisms such as subclinical atrial cardiomyopathy [2]. Data on AFL prevalence by race are limited. Often, authors combine AF and AFL into a single entity, perhaps reflecting the historical belief that the two arrhythmias exist on a continuum, or to simplify analysis. Among patients with pacemakers, where continuous monitoring minimizes ascertainment bias, Black patients demonstrate a lower hazard of AF but a higher hazard of isolated AFL; nonetheless, the composite burden of AF or AFL remains lower in Black patients compared to White patients [3].

Catheter ablation (CA) has emerged as an effective therapeutic strategy for both AF and AFL, with recent clinical practice guidelines elevating CA to a first-line consideration, particularly for patients with heart failure [4]. Recent advances in mapping technology, contact force-sensing catheters, and novel non-thermal energy source technology, have increased clinician confidence in considering CA as a primary rhythm control strategy. Indeed, there was a steady increase in the number of AF ablations performed in the United States in the years leading up to the 2023 ACC/AHA/ACCP/HRS guideline changes regarding the diagnosis and management of atrial fibrillation [4], with an even steeper rise following the update [5, 6]. However, catheter ablation is used more commonly in AFL than AF likely due to the inherently high success rates of CA for AFL [7]. Dedicated studies examining CA utilization disparities specifically for AFL remain sparse. One of the few AFL-specific analyses using the National Inpatient Sample (NIS) reported that racial and ethnic minorities are less likely to undergo catheter ablation for AFL compared to White patients [8]. Despite having Class I indications, Black and Hispanic patients with AF and typical AFL receive lower rates of CA compared to White patients [7, 9, 10].

In a North American subgroup analysis of the landmark CABANA trial, racial and ethnic minority patients randomized to CA experienced a 68% relative reduction in the primary composite endpoint (aHR: 0.32, 95% CI: 0.13–0.78) and a 72% relative reduction in all-cause mortality (aHR: 0.28, 95% CI: 0.10–0.79) compared to those on drug therapy [11]. These findings, however, must be interpreted with caution as the minority subgroup analyzed comprised only 127 of the 1,280 North American participants, and this analysis was post-hoc and hypothesis-generating rather than pre-specified. The magnitude of benefit was largely driven by exceptionally poor outcomes among minoritized patients under anti-arrhythmic drug therapy, which suggests heightened baseline risk rather than differential procedural efficacy per se. Furthermore, racial and ethnic minorities were underrepresented in the trial at baseline, reflecting a broader pattern identified across major AF trials in which fewer than half report participant-level race/ethnicity data [12]. Despite these important caveats, the CABANA subgroup data illustrate the treatment-risk paradox most consequentially: the populations bearing the greatest burden of adverse outcomes under standard therapy are precisely those least likely to access the intervention that might benefit them the most.

This state-of-the-art review synthesizes existing evidence on the racial/ethnic distribution of CA utilization for both AF and AFL in the United States to identify gaps in the literature and propose a path forward for equitable invasive AF/AFL care. We further explore the multifaceted nature of the racial disparity in the utilization of CA and discuss mechanisms that may explain the observations.

## Conceptual Framework

**Fig. 1:**
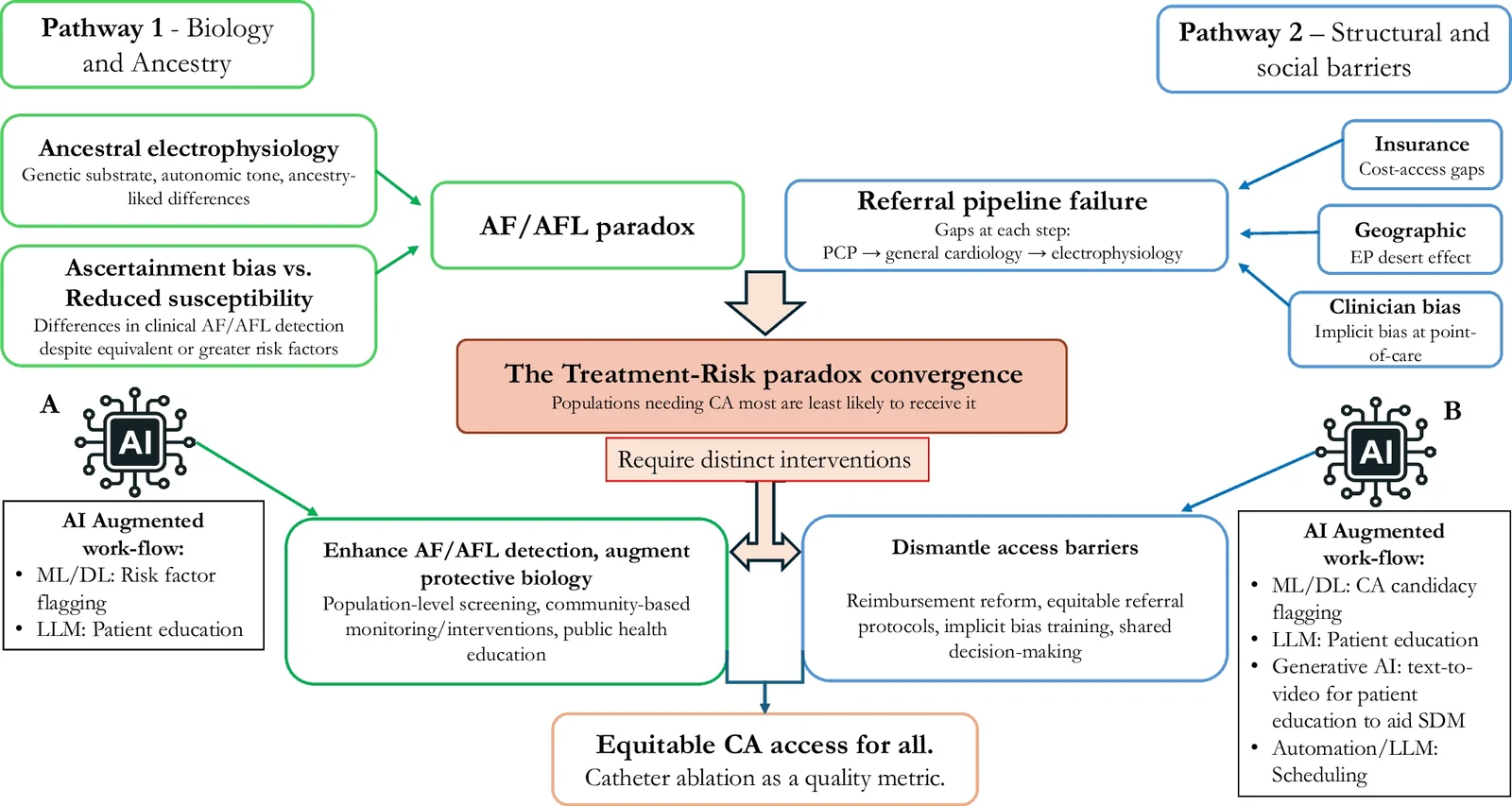
Dual-pathway conceptual framework: AF/AFL prevalence paradox versus catheter ablation access disparity. Pathway 1: Ancestry-linked biological differences drive the AF/AFL prevalence paradox. Pathway 2: Structural and social barriers drive the CA access disparity. Artificial intelligence (AI)-augmented work-flows may help reduce blind spots to bridge the detection and access gaps. **A**. This sequential framework utilizes machine learning (ML) and deep learning (DL) models to analyze multi-source clinical data and automatically flag individuals at elevated risk for AF/AFL. Following clinician validation, the pipeline deploys a large language model (LLM) to synthesize specific risk factors into personalized, highly accessible educational text that empowers the patient to pursue targeted lifestyle modification and preventative care. **B**. This framework utilizes ML and DL to analyze unstructured ECG, echocardiographic or MRI data and automatically flag high-burden patients meeting criteria for CA. Following clinician validation, the pipeline orchestrates an LLM, text-to-video generative AI, and EHR-integrated automation to seamlessly translate complex medical concepts into accessible multi-lingual educational media, enhance shared decision making (SDM) and schedule the procedure. Abbreviations: AF = atrial fibrillation; AFL = atrial flutter; CA = catheter ablation; EP = electrophysiology; ECG = electrocardiogram; PCP = primary care provider; AI = artificial intelligence; ML = machine learning; DL = deep learning; LLM = large language model; MRI = magnetic resonance imaging; EHR = electronic health records; SDM = shared-decision making

Throughout this review, we use “Black” as a social construct, consistent with contemporary cardiovascular literature, recognizing that it encompasses individuals of African descent across diverse national and cultural contexts. We distinguish this from ancestry-based or continental designations used in epidemiologic studies of AF prevalence where genetic and electrophysiologic factors may be more relevant. This distinction is not merely semantic, as it reflects fundamentally different causal pathways underlying the AF/AFL prevalence paradox on one hand, and the disparities in access to advanced arrhythmia care on the other. We propose a dual-pathway conceptual framework as the organizing thesis of this review and suggest that it should be adopted to guide epidemiologic arrhythmia research. We postulate that for AF/AFL prevalence, ancestry and biology matter; hence the paradox. However, for advanced invasive therapy access and utilization, race/ethnicity as a social construct is the operative variable; hence the disparity. These pathways are structurally distinct and must not be conflated in research design or clinical interpretation. The AF prevalence paradox reflects differences in electrophysiologic substrate, autonomic tone, genetic and epigenetic architecture across ancestral groups. The CA access disparity, in contrast, reflects the downstream consequences of structural barriers, differential resource distribution, and institutional bias operating through the healthcare system. Collapsing both phenomena into a single regression framework, as most existing studies do, risks attributing to biology what is in fact a product of social structure, and vice versa. This framework is illustrated in Fig. 1.

This conceptual framing carries direct implications for how disparities in CA should be studied, understood and addressed. First, future research must move beyond merely documenting that disparities exist toward identifying the structural mechanisms, including insurance status, referral patterns, geographic access, and implicit bias, which sustain them. Second, clinical interventions aimed at closing the ablation gap must target the social and systemic determinants of access rather than attribute underutilization to patient-level factors such as preference or comorbidity burden alone, as the latter risks mislocating the problem and allowing structural failures to persist. Third, guideline-writing bodies and professional societies must treat equitable CA access as a measurable quality indicator, recognizing that a therapy elevated to first-line status carries an ethical obligation of equitable distribution across all racial and ethnic groups.

## Disparities in Catheter Ablation for Atrial Fibrillation

Two recent independent meta-analyses, both with literature searches extending through 2024, synthesized the literature on racial and ethnic disparities in CA utilization. The first identified 18 eligible studies and demonstrated significantly lower CA utilization in Black, Hispanic, and Asian patients compared to White patients across multiple database sources and study designs. Black patients had an adjusted odds ratio of 0.65 (95% CI: 0.58–0.74) of undergoing CA compared to White patients [13]. Similarly, Hispanic patients showed reduced odds of CA (aOR: 0.78; 95% CI: 0.73–0.83), while Asian patients had an aOR of 0.74 (95% CI: 0.54–1.0). The second meta-analysis included 19 studies and reported a higher proportion of CA among White individuals (4%) compared to Black, Hispanic, and Asian patients (each approximately 2%). In pooled analyses, the odds of receiving CA were 0.68 (95% CI: 0.56–0.83) for Black individuals, 0.72 (95% CI: 0.63–0.82) for Hispanic individuals, and 0.64 (95% CI: 0.48–0.86) for Asian individuals compared to White individuals [14].

These pooled estimates must be interpreted with substantial caution. Between-study heterogeneity was extreme across both meta-analyses, with *I*^*2*^ values ranging from 78 to 99%. At these levels, a single pooled estimate is statistically incompatible with the underlying data and cannot be considered a reliable summary statistic. The high degree of heterogeneity across these analyses suggests that the underlying mechanisms of disparity may vary substantially based on source population, study methodology, and risk adjustment. In fact, the level of heterogeneity in these meta-analyses greatly limits their utility in providing reliable quantitative estimates of the disparity magnitude. Instead, they serve as a catalog of the literature and its limitations, and indicate the need for properly structured investigation of disparities in CA access. Notably, our review of the literature indicates that conference abstracts and administrative data analyses form the bulk of studies addressing this very important question.

Several all-payer data studies report significantly lower rates of AF ablation among Black, Asian and Hispanic patients in the inpatient setting [15, 16]. In an analysis of hospitalizations for symptomatic AF across California, New York and Florida, White patients accounted for the vast majority of the procedural cohort undergoing CA, at 83.9% [15]. Conversely, racial and ethnic minorities were underrepresented, with Hispanic patients making up 6.9%, Black patients 5.5%, and Asian patients 1.6% of those who received CA. Even after adjusting for clinical comorbidities, insurance type and median income, multivariate analysis demonstrated that the likelihood of undergoing CA was significantly lower for Hispanic (HR = 0.60), Asian (HR = 0.58) and Black patients (HR = 0.68) compared to White patients. These marked racial disparities remained despite minoritized populations experiencing similar or higher rates of subsequent AF-related hospitalization [15]. Patel and colleagues reported similar results using the NIS from 2000 through 2012. In that study, the odds of undergoing CA were 0.49 for Black patients and 0.64 for Hispanic patients compared to their White counterparts despite a 940% increase in the number of ablations over the study period [16].

## Disparities in Atrial Flutter Ablation Utilization

An obvious data deficit persists in the context of CA utilization for stand-alone AFL. A study using the NIS reported a two-and-a-half fold increase in the volume of CA for AFL from 2000 to 2011 [17]. Of the 89,638 encounters for CA included in that analysis, 64.5% were White patients while Black and Hispanic patients represented 7% and 3.1%, respectively. The study did not report rates among Asians or Native Americans and 22.3% of racial data was missing. Another study by Tamariz et al. focused on patients who sought care in Florida, analyzing 28,714 hospital encounters for stand-alone AFL catheter ablation [7]. The investigators revealed that Black patients had significantly lower odds of undergoing the procedure compared to White patients (OR 0.72, 95% CI: 0.58–0.88), a disparity that was further compounded among female minority patients. This deficit in the utilization of CA for stand-alone AFL highlights a serious clinical oversight as AFL ablation boasts an excellent safety profile and immediate success rates of 90–95% [18]. Given this risk–benefit profile, it is difficult to attribute the underutilization of CA for AFL in minoritized cohorts to patient or clinician perception of poor benefit or excessive complexity. Instead, the persistent disparity is more consistent with systemic access barriers than with clinical hesitation or patient-level risk factors.

## Disparities in Ambulatory Ablation Procedures

Recent trends toward same-day discharge and ambulatory AF ablation represent a significant shift in procedural delivery models. This innovation has not benefited all populations equally. Data from the Nationwide Ambulatory Surgery Sample showed that despite an appreciable increase in procedure volume, racial disparities in CA utilization rates persisted in this setting as well. Across both sexes, White individuals were 2.9 to 3.8 times more likely than individuals of any racial group to receive CA over the 3-year study period [19]. Ambulatory center ablations and same-day discharge procedures are more likely to occur in lower-comorbidity patient populations. Therefore, comorbidity burden would not explain the observed disparity in utilization rates. These findings suggest that the shift towards ambulatory and same-day discharge practice models, while broadly beneficial, has not been equitably distributed and may be compounding pre-existing racial gaps in procedural access.

## Differences in Post-Ablation Outcomes

Racial disparities extend beyond utilization to encompass procedural safety and immediate outcomes. In an analysis of 94,245 hospitalized encounters involving patients with a history of congestive heart failure and undergoing CA for AF using the NIS (2016–2021), Black patients were noted to be younger (64 years versus 71 years) but experienced higher rates of periprocedural complications including cardiogenic shock (7.8% versus 5.6%, *p* < *0.005*) and major adverse cardiovascular/cerebrovascular events (12.6% versus 9.9%, *p* < *0.005*) when compared to White patients [20].

Using real-world data from TriNetX, Sinha and colleagues showed a rare but important post-ablation complication, stress-induced cardiomyopathy (Takotsubo syndrome, TS), which was higher in minoritized groups [21]. Black patients comprised 7.4% of the post-ablation TS cases despite making up just 5.1% of the baseline ablation cohort. While the small sample size (27 TS cases) precluded this specific incidence discrepancy from reaching statistical significance, a clear racial disparity emerged in overall 30-day mortality across the entire ablation cohort: upon multivariable adjustment, Black patients experienced significantly higher odds of 30-day post-ablation mortality compared to White patients (OR = 1.78, 95% CI:1.02–3.11, *p* = *0.04*).

These findings raise the concern that when Black patients do undergo CA, they may do so later in their disease course or with heavier comorbidity burden, which shows the downstream effects of the access barriers discussed above, rather than evidence that ablation itself is a poor choice for minoritized patients. This distinction matters because the long-term benefits of CA, when access is not delayed can be substantial: long-term data post-CA for AF show significant reduction in ischemic stroke events (7.9% versus 9.5%, *p* < *0.001*) compared to those who did not have CA [22]. Similarly, all-cause mortality at 5 years was lower in the CA group (9.3% versus 20.7%, *p* < *0.001*). Unfortunately, this study did not stratify outcomes by race; the authors did, however, report a significantly higher proportion of Black, Hispanic, and Asian patients did not undergo CA.

Taken together, catheter ablation for AF is associated with robust long-term reduction in meaningful outcomes, but the lower utilization rates among minoritized patients present a source of excess morbidity and mortality, which calls for careful attention to efforts to dismantle the structural and systemic barriers that fuel cardiovascular care inequity.

## Disparities in AF-related Outcomes

Once diagnosed with AF, Black patients suffer a disproportionately higher burden of adverse cardiovascular events. Epidemiological data demonstrate that Black patients with AF face significantly elevated risk for ischemic stroke, coronary artery disease, heart failure, and all-cause mortality compared to their White counterparts [23]. This divergence in outcomes highlights critical racial disparities in arrhythmia-related morbidity and mortality, particularly given the differential impact of the AF paradox [24]. Notably, the higher hazard of ischemic stroke in Black patients with AF is often not directly driven by the arrhythmia itself, but rather by a higher prevalence of uncontrolled comorbidity profiles such as severe hypertension, left ventricular hypertrophy, and chronic kidney disease. Further, these clinical disparities are then compounded by systemic inequities in healthcare access [25]. Black patients are frequently underrepresented in rhythm control strategies, face lower rates of anticoagulant prescription and adherence, and experience delays in referral to electrophysiology (EP) specialists, ultimately accelerating adverse disease progression.

## Structural Mechanisms Underlying the Treatment-Risk Paradox in CA Utilization

Structural barriers may contribute to and sustain inequities in health care access. To dismantle these barriers in CA utilization, the cardiac EP community must move beyond mere documentation of disparities and instead delineate the specific structural mechanisms that govern the path from an initial arrhythmia diagnosis to the EP laboratory. These mechanisms operate at the macro-systemic, geographic, and institutional levels. We consider each in turn, from policy-level forces shaping insurance and referral patterns to the institutional-level factors that determine who ultimately reaches the EP laboratory.

## Insurance Design, Financial Burden, and Systemic Factors

Out-of-pocket costs and structural healthcare coverage discrepancies heavily influence utilization disparities in CA access. As a highly resource-intensive procedure with significant upfront institutional costs, CA utilization is shaped by policy-level, institutional and patient-level barriers that compound across the care pathway. At the policy-level, Medicaid reimbursement models for highly technical procedures such as CA vary drastically by state and frequently trail private payer rates, disincentivizing safety-net institutions from building robust electrophysiology programs. This payer-driven disinvestment shapes institutional referral patterns. Data consistently demonstrate that patients with commercial or private insurance are far more likely to undergo CA than those relying on Medicaid or public assistance [26, 27]. Even among insured patients, out-of-pocket costs introduce a final and patient-level barrier: the co-pays associated with invasive rhythm control may deter low-income and minoritized patients from consenting to CA, funneling them instead to less effective, long-term antiarrhythmic drug regimens.

## Referral Pipelines and Geographic Distribution of Electrophysiology Services

The primary structural bottleneck in CA utilization resides within the upstream referral pipeline. Patients do not self-refer for catheter ablation; they rely on a tightly gated sequence moving from primary care clinicians or the emergency department to a general cardiologist, and ultimately to a cardiac electrophysiologist. An analysis of Medicare beneficiaries showed that minoritized patients are significantly less likely to be evaluated by a general cardiologist when presenting with new-onset AF or AFL, which heavily compromises downstream referral to EP specialists [28]. Duke and colleagues investigated whether differences in referral patterns played a role in the racial disparity in CA utilization for AF using data from a large healthcare system in Chicago. Interestingly, even within this integrated health system where minoritized patients were referred to general cardiology at equal or higher rates, systemic fractures still occurred downstream. Black patients who successfully navigated the pipeline to be evaluated by an EP specialist were 37% less likely to undergo CA compared to their White counterparts [29]. This reveals a dual-layered barrier: a macro-level deficiency in specialty care access compounded by a structural failure occurring directly within electrophysiology consultation space itself.

Independent of referral behavior within existing systems, geographic access acts as a second structural determinant of care. Racial and ethnic minority populations are disproportionately clustered in resource-constrained geographic areas or serviced by safety-net hospital systems that lack dedicated EP laboratories. This creates a baseline structural barrier of geographic distance and regional scarcity layered atop the broader cardiology deserts already documented in the literature: what might be termed an *EP desert*. Indeed, contemporary geographic analyses demonstrate that one-third of Black Americans reside in counties classified as cardiology deserts, where access to basic cardiovascular care already requires extensive travel times [30, 31]. Given that electrophysiology centers are even more tightly clustered within affluent, metropolitan, or academic hubs, this regional scarcity is profoundly amplified for advanced arrhythmia care.

## Clinician-Level Implicit Bias and Clinical Decision-Making

At the point of clinical contact, implicit bias among clinicians operates as a subtle yet powerful filter. Historical health disparity literature has inadvertently mischaracterized the utilization gap by pointing to “patient preference”, suggesting that minoritized populations are more averse to invasive cardiac procedures. While historical healthcare injustices suffered by minoritized populations, especially Black patients, have contributed to the well-documented distrust of medical institutions and subsequent delays in seeking medical care, the assumption that decision inertia on the part of minoritized patients underpins the lower utilization rates of CA is an oversimplification of a complex problem. Contemporary health equity research suggests that clinical decision-making is heavily influenced by subjective clinician perceptions regarding a patient’s social support network, health literacy, compliance capacity, and ability to manage post-procedural care or strict anticoagulation regimens [32].

Two landmark systematic reviews provide the empirical basis for understanding implicit bias as a clinical mechanism. FitzGerald and Hurst examined 42 studies and demonstrated that healthcare professionals exhibit implicit bias toward patients at rates comparable to the general population, and that these biases influence clinical treatment decisions and patient-clinician interactions [33]. Independently, Hall and colleagues conducted a systematic review of 15 studies and reported that most healthcare workers harbor implicit pro-White bias as measured by the Implicit Association Test, and that these attitudes are more often associated with patient-clinician interactions and health outcomes than with consciously expressed treatment preferences [34]. In the cardiovascular context specifically, these biases may manifest as differential thresholds for offering invasive procedures. If an electrophysiology clinician implicitly perceives a minoritized patient as less likely to comply with post-ablation follow-up or anticoagulation regimens, they may be less inclined to offer CA as a first-line option. This subjective risk assessment effectively shifts the burden of systemic failures onto the patient, creating an artificial barrier to a Class I-indicated therapy.

Rather than reflecting intrinsic patient aversion, gaps in procedural utilization frequently stem from a breakdown in clinician-led counselling and the omission of structured shared decision-making (SDM). Contemporary data in AF management demonstrate that structured SDM remains severely underutilized; in a large cohort of patients pursuing rhythm control strategies, only 22% reported participating in an SDM process, while approximately half reported providing no input into their treatment plan at all [35]. Crucially, patients excluded from SDM were significantly less likely to understand their rhythm control options [35]. This highlights that patient unfamiliarity with a highly technical procedure like CA is a downstream consequence of inadequate, non-standard clinician communication rather than an independent patient-side variable. Implementing a culturally sensitive, proactive SDM framework within the EP consultation space is therefore a structural necessity to repair these communication fractures and ensure equitable access.

## Current Gaps in the Literature

When large administrative datasets are leveraged to examine AFL ablation utilization and outcomes, racial data is frequently relegated to a baseline demographic variable or treated merely as a covariate for multivariate adjustments (e.g. [36]). While statistically necessary to isolate the independent therapeutic effect of ablation on healthcare utilization, this practice inadvertently conceals granular, stratified rates of ablation access, acute procedural success, and long-term complication rates across distinct racial and ethnic cohorts. This lack of granularity creates a significant clinical/epidemiological blind spot. Stand-alone AFL carries a distinct pathophysiological profile, remodeling pattern and stroke risk compared to mixed AF/AFL phenotypes. By failing to report stratified ablation rates and long-term outcomes by race/ethnicity, the electrophysiology community remains unable to identify potential race-based biases in referral patterns or post-procedural disparities unique to AFL care.

The National Cardiovascular Data Registry (NCDR) AFib Ablation Registry initiated by the American College of Cardiology in part to assess the continually changing landscape of AF ablation, represents a major paradigm shift in our structural capacity to study health equity in cardiac electrophysiology. Unlike administrative databases such as the NIS which suffer from crude, historically rigid demographic categories, the NCDR data dictionary early on adopted forward-thinking, granular classifications. Crucially, this included the distinct Middle Eastern or North African (MENA) category and a dedicated Hispanic/Latino classification, as well as subgroups for Asian identity including Indian, Chinese, Korean, Japanese, Vietnamese, and Filipino. This granular architecture provides an unprecedented framework for deliberate analysis of ablation utilization for both AF and AFL (as distinct or mixed presentations), and their associated outcomes among precisely defined cohorts.

Despite this sophisticated data collection infrastructure, a stark implementation gap persists in the published literature. Early benchmark studies utilizing the NCDR AFib Ablation Registry such as reported by Hsu and colleagues, continue to report heavily aggregated data [6]. The study revealed that cohort is over 94% White, and no race-stratified outcome analysis was presented. This mirrors the broader pattern documented by Sarraju and colleagues, which found that fewer than half of trials informing the 2019 AHA/ACC/HRS AF guidelines reported participant-level race/ethnicity data [12]. This systemic under-reporting renders the evidence base for AF management poorly generalizable to the minoritized populations who bear a disproportionate burden of arrhythmia-related morbidity. By failing to leverage the registry’s unique data elements, current clinical literature inadvertently perpetuates the demographic blind spots characteristic of older, less refined datasets.

Closing this implementation gap will require the field to fully operationalize the granularity the NCDR already collects. To that end, health epidemiology and electrophysiology research communities must universally adopt and mandate the collection of granular data utilizing the updated White House Office of Management and Budget (OMB) Statistical Policy Directive 15 standards [37]. By standardizing data collection around these revised seven minimum categories (including the official addition of the MENA category and the structural shift to a single combined race and ethnicity question format), researchers can eliminate the artificial aggregation that obscures distinct cohorts.

This recommendation is critically tethered to a shifting federal regulatory landscape. Following the March 2024 OMB policy directive, federal agencies including the Centers for Medicare & Medicaid Services face strict compliance timelines, with full implementation required by March 2029. Operationalizing this mandate will require electronic health record systems to adopt the updated architecture at the point of care. This will be a vital link in translating federal administrative policy into high-resolution health equity research.

## Methodological Recommendations for Future Research

To rectify this deficit and fully realize the potential of registries such as the NCDR, the electrophysiology research community should consider adopting the following practices. First, future registry-based investigators should shift away from using race merely as a baseline variable for covariate adjustment. Instead, studies must explicitly report race/ethnicity-stratified analyses to unmask hidden disparities in procedural efficacy, safety, and post-ablation care. Second, for health equity and clinical outcomes research in AF/AFL ablations, investigators should favor detailed registries over administrative claims databases. Claims data offer large sample sizes but lack the granularity such as procedural detail, longitudinal follow-up, and clinical context, needed to meaningfully examine disparities in care.

Third, future studies should adopt a theoretically grounded framework for disparity analysis. The Institute of Medicine definition of disparity — which identifies as unjust any differences in care quality not explained by health needs, clinical appropriateness, or patient preference — provides the most rigorous conceptual anchor and should be adopted as the standard definition [38, 39]. Under this framework, variables such as socioeconomic status and social determinants of health should be treated as mediators of structural barriers to access rather than confounders to be eliminated, as adjusting for them obscures rather than illuminates the causal pathways driving inequity.

Fourth, and most critically, future studies should incorporate adjustment for the clinical variables that legitimately govern CA candidacy in order to better distinguish true inequity from appropriate clinical variation. Per the 2023 ACC/AHA/ACCP/HRS guideline, clinical candidacy is primarily driven by symptom status, the formalized AF stage, and specific echocardiographic or clinical comorbidities, most notably, the presence of heart failure with reduced ejection fraction [4]. Omission of these clinical determinants from disparity models risks conflating clinically appropriate variation with inequitable access. However, even with adjustment, residual disparity estimates remain vulnerable to unmeasured confounders such as access barriers, unmeasured social determinants of health, or unrecorded clinical factors, and should be interpreted accordingly. Explicit adjustment for these variables will enable a more meaningful, albeit imperfect, separation of the two. Without these methodological standards, disparity estimates will remain both unreliable in magnitude and uninformative about mechanisms, which will undermine their utility for designing targeted interventions and informing policy change.

## Conclusion and a Path Forward

The evolution of AF/AFL care, marked by newer technologies and guideline updates signifies progress in modern arrhythmia care. However, as shown in this review, the fruits of this advancement are not equitably distributed. The survival and secondary stroke prevention benefits of CA, suggested by the CABANA minority subgroup analysis and supported by real-world outcomes studies, are not consistently being used by the very minoritized populations who suffer the worst clinical outcomes under standard medical therapy.

The central thesis of this review is that the AF/AFL prevalence paradox and the CA access disparity represent two structurally distinct phenomena operating through different causal pathways. The prevalence paradox is anchored in ancestry and biology, while the access disparity is anchored in race as a social construct. These phenomena contribute to the disparity in AF/AFL care and require different interventions to overcome. The paradox calls for enhance population-level detection in addition to exploring novel biological mechanisms that govern the differential conversion of risk factor accumulation to clinically detectable arrhythmia, while the access disparity calls for systemic accountability in how CA is offered, referred, and delivered.

Resolving CA access inequity requires an aggressive pivot from descriptive epidemiology to systemic accountability. Research must transition toward publishing race-stratified registry or longitudinal data, standardizing equitable referral protocols across health systems, and treating equitable procedural access as a core institutional metric of clinical quality. Professional societies must recognize that elevating CA to first-line therapy without simultaneously mandating equitable access creates a new vector for health inequality: the guideline becomes the standard only for those who can access the procedure.

Emerging artificial intelligence-augmented workflows offer a promising, scalable complement to these structural reforms [40]. Machine learning algorithms embedded within the electronic health record can potentially flag patients meeting guideline-based CA candidacy criteria who have not yet been referred. This may directly counteract the referral pipeline fractures described above rather than waiting for clinician-initiated recognition. Natural language processing tools may further standardize patient education by generating culturally tailored, literacy-appropriate materials on CA at the point of care, addressing the communication breakdown that underlies much of the misattributed “patient preference” narrative. Finally, AI-driven scheduling systems capable of proactively identifying and triaging high-risk, under-resourced patients could help ensure that finite electrophysiology laboratory capacity is allocated equitably rather than reproducing legacy referral patterns. These tools carry their own risks of encoding and perpetuating existing biases if trained on historically inequitable data, and must therefore be developed and audited with explicit attention to equitable performance across racial and ethnic subgroups [41]. Nonetheless, a thoughtfully implemented AI augmentation strategy represents a novel structural lever, alongside policy and institutional reform, for closing the gap between guideline eligibility and real-world CA delivery.

Only by addressing the structural determinants of access, through reimbursement reform, geographic redistribution of electrophysiology services, implicit bias training at the institutional level, AI-augmented candidacy identification, and culturally informed shared decision-making, can we ensure that these life-saving technological advancements serve to close, rather than widen, the equity gaps in invasive arrhythmia care.

## Key References

- Nosair, W., Shaban, L., El Khoury, M., Al Ashqar, R., Heimlich, L., Kolm, P., Smith, J., Dolman, S., Tsimploulis, A., Thomaides, A., Strouse, D., Lee, S., Weintraub, W. S., & Mongraw-Chaffin, M. (2025). Racial and ethnic disparities in catheter ablation utilization for atrial fibrillation. *JACC: Advances*, *4*(9), 102105. https://doi.org/10.1016/j.jacadv.2025.102105
  - ○ This study shows that non-Hispanic Black, Hispanic/LatinX, and Asian patients are significantly less likely to receive catheter ablation for atrial fibrillation compared to non-Hispanic White patients.
- Moinul, S., Urina-Jassir, M., Rodriguez-Taveras, J., Peralta, A. O., Hoffmeister, P. S., Kinlay, S., Yarmohammadi, H., Boden, W. E., Joseph, J., & Yuyun, M. F. (2025). Meta-analysis of racial and ethnic disparities in rhythm control strategies for atrial fibrillation in the United States. *The American Journal of Cardiology*, *245*, 1–10. https://doi.org/10.1016/j.amjcard.2025.02.028
  - ○ This study found that racial and ethnic minority groups with atrial fibrillation are less likely to undergo rhythm control strategies including catheter ablation compared to White patients.

## Data Availability

No datasets were generated or analysed during the current study.
